# Recent molecular approaches to understanding astrocyte function *in vivo*

**DOI:** 10.3389/fncel.2013.00272

**Published:** 2013-12-24

**Authors:** David Davila, Karine Thibault, Todd A. Fiacco, Cendra Agulhon

**Affiliations:** ^1^Glia-Glia and Glia-Neuron Interactions Group, National Center for Scientific Research, UFR Biomedicale, Paris Descartes UniversityParis, France; ^2^Department of Cell Biology and Neuroscience, and Center for Glial-Neuronal Interactions and Program in Cellular, Molecular and Developmental Biology, University of California at RiversideRiverside, CA, USA

**Keywords:** astrocytes, knockout mice, transgenic mice, chemogenetics, viral gene transduction, glial cell progenitors, GPCR, neuron–glia interactions

## Abstract

Astrocytes are a predominant glial cell type in the nervous systems, and are becoming recognized as important mediators of normal brain function as well as neurodevelopmental, neurological, and neurodegenerative brain diseases. Although numerous potential mechanisms have been proposed to explain the role of astrocytes in the normal and diseased brain, research into the physiological relevance of these mechanisms *in vivo* is just beginning. In this review, we will summarize recent developments in innovative and powerful molecular approaches, including knockout mouse models, transgenic mouse models, and astrocyte-targeted gene transfer/expression, which have led to advances in understanding astrocyte biology *in vivo* that were heretofore inaccessible to experimentation. We will examine the recently improved understanding of the roles of astrocytes – with an emphasis on astrocyte signaling – in the context of both the healthy and diseased brain, discuss areas where the role of astrocytes remains debated, and suggest new research directions.

## INTRODUCTION

Although astrocytes are the most abundant glial cell type in the mammalian nervous system and have emerged as crucial regulators of nervous system development, function, and health, our understanding of the physiology of astrocytes remains limited. How do astrocytes interact with neurons and other cell types of the nervous system? What are the primary functions of astrocytes in brain development, health, and disease? One challenge to address these questions is identifying the function of individual astrocytic molecules that regulate brain function. A unique approach to investigate the molecular basis of astrocyte activity consists of manipulating the genome of higher organisms. The mouse represents a great animal model that has been used extensively for genetic manipulation in neuroscience, leading to understanding of neuronal functions in unprecedented detail. A concerted effort in recent years to develop genetic approaches to study astrocytes is guiding the field into new territory and improving our understanding of interactions between neurons and astrocytes. Here we will review techniques for genetic manipulation of astrocytes and highlight the most recent innovative and elegant approaches that are providing insight into fundamental roles of astrocytes in pathophysiology *in vivo*. In particular, we will describe the main molecular approaches used in this field, including knockout (KO) mouse models, transgenic mouse models, and astrocyte-targeted gene transfer and expression using adeno-associated viral (AAV) or *in utero* electroporation (IUE) approaches. We will also touch on a remarkable recent study involving engraftment of genetically modified human glial progenitors into the mouse brain, providing insight into the role of human astrocytes in the unique cognitive abilities of the human brain. The present review does not attempt to be comprehensive; rather it highlights certain major themes and areas of recent progress on the roles of astrocytes in brain function, with an emphasis on astrocyte signaling and *in vivo* studies. For complementary reviews on astrocyte function in health and disease with an emphasis on molecular approaches, readers are directed to (Fiacco et al., 2009b; Figueiredo et al., 2011; Agulhon et al., 2012; Nedergaard and Verkhratsky, 2012; Clarke and Barres, 2013; Freeman and Rowitch, 2013; Tong et al., 2013), as well as the other contributions to this special topic.

## STUDYING ASTROCYTE FUNCTION THROUGH GENE DISRUPTION

The elimination of one or more specific genes in an animal model is a reliable and widespread approach to discovering the function of specific proteins and the cell types expressing them. Many genes have been identified, isolated, and subsequently manipulated to fully or conditionally suppress their expression (Sikorski and Peters, 1997; Grimm, 2006; Vogel, 2007). These molecular developments represent powerful tools that can be used *in vivo* to aid in discovering the role of astrocytes in the healthy and diseased brain.

### FULL (CONSTITUTIVE) KNOCKOUT MOUSE MODELS

**FIGURE 1:**
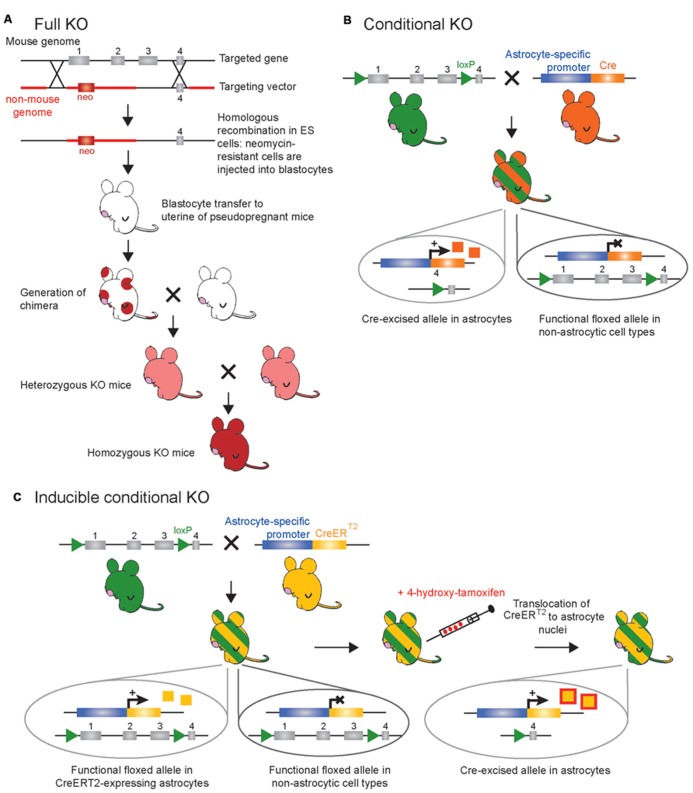
**Schematic representation of the main genetic manipulations to make knockout (KO) mice.** **(A)** Conventional strategy for full (constitutive) gene knockout. The targeted gene is inactivated following insertion of an antibiotic (neomycin or neo) resistance gene within, or in place, of one of several essential exons(s) through electroporation of a targeting vector in murine embryonic stem (ES) cells and subsequent homologous recombination. Totipotent embryonic ES cells with successful recombination will survive neomycin treatment to be selectively chosen and injected in mouse blastocysts. Blastocytes are then transferred to the uterus of pseudopregnant females where they can differentiate into all cell types of a chimeric mouse (red circles). After breeding the chimeric mice, the resulting offspring will derive from the ES cells – as seen with the transmission of coat color (red) – if the introduced ES cells become established into the germline of the chimeric mouse. In the full KO, the gene of interest is knocked out in all cell types (not only astrocytes) of the offspring. **(B)** Classical Cre-*LoxP* strategy for conditional gene knockout. The creation of two mouse lines is necessary. First, a floxed mouse line (green) is obtained by homologous recombination in ES cells. Two *LoxP* sites are positioned in intronic regions that flank one or several essential exons of the targeted gene. In this mouse line the targeted gene is normally expressed. Second, a Cre mouse line (orange) created by classical transgenesis, following pronuclear injection of the cDNA encoding the Cre-recombinase under the control of an astrocyte-specific promoter. This line gives astrocyte selectivity to the system. Breeding of the floxed mouse with the Cre mouse leads to the generation of a new mouse line (green/orange-striped) in which the floxed exon(s) are excised only in astrocyte-expressing Cre, while the targeted gene remains functional in other cell types. In this case the Cre-recombinase (orange squares) is constitutively expressed, i.e., inactivation of the targeted gene occurs as soon as the astrocyte-specific promoter driving Cre-recombinase is active. **(C)** Inducible CreER^T2^-*LoxP* strategy for temporal control of gene knockout. As in **(B)**, a floxed mouse line (green) and a CreER^T2^ mouse line (yellow) are necessary to obtain a third mouse line (green/yellow-striped) in which the floxed exon(s) are excised only in astrocyte-expressing CreER^T2^. In this mouse line, the CreER^T2^-recombinase (yellow squares) is expressed in astrocytes but is kept in the cytoplasmic compartment and is inactive. To achieve temporal control of the gene knockout, CreER^T2^ activity (red/yellow squares) is induced by synthetic steroid ligand (tamoxifen or 4-hydroxy-tamoxifen capable of crossing the blood brain barrier) administrated systemically at any chosen time. Binding of steroid to CreER^T2^ allows translocation of CreER^T2^ to the nucleus where recombination of floxed genes can occur selectively in astrocytes while the targeted gene remains functional in other cell types.

As research into astroglia physiology *in vivo* is still a newly developing field, much information can be gleaned from the elimination of a gene or the deletion of a functional domain of a protein in astrocytes to shed light on the function of both the targeted gene and astrocytes in general. The process of generating a new line of KO mice is laborious, but has been refined to maximize efficiency (reviewed in Hall et al., 2009; Limaye et al., 2009). Once the desired gene is identified, gene targeting can be used to generate a KO mouse. A targeting vector containing a neomycin-resistant marker is inserted into embryonic stem (ES) cells via electroporation and is introduced into the DNA through homologous recombination, allowing complete removal of one or more exons from the gene of interest (**Figure 1A**). This results in the production of a mutated or truncated protein or, more often, no protein at all. ES cells that do not take up the foreign construct are killed through exposure to neomycin, and those that have successfully replaced the gene or the exons of this gene survive and are subsequently microinjected into mouse blastocysts, which are then grown in surrogate mouse uteri. Strategic mating of the chimeric mice will ultimately result in a mouse with the gene globally eliminated (Capecchi, 1989; Hall et al., 2009; Limaye et al., 2009; **Figure 1A**).

#### Role of GFAP in the healthy and diseased brain

One of the first genes that was removed in astrocytes is the gene encoding glial fibrillary acidic protein (GFAP; Lewis et al., 1984; Reeves et al., 1989; Masood et al., 1993; McCall et al., 1996; Eng et al., 2000). GFAP is a member of the family of intermediate filament structural proteins, which, in the mature nervous system, is found predominantly in protoplasmic and specialized astrocytes of the central nervous system (CNS) as well as in satellite cells, non-myelinating Schwann cells, and enteric glia in the peripheral nervous system (PNS; Jessen et al., 1984; Eng, 1985; Kato et al., 1990). Outside the nervous system, GFAP has also been detected in some rare non-glial cells of the salivary glands (Achstatter et al., 1986; Gustafsson et al., 1989), fibroblasts (Hainfellner et al., 2001), myoepithelial cells (Viale et al., 1991), liver stellate cells (Gard et al., 1985), and lymphocytes (Riol et al., 1997). This protein is one of the key elements of the cytoskeleton that contributes to the morphology and motility of astrocyte processes (Fuchs and Weber, 1994; Pekny and Pekna, 2004; Gomi et al., 2010; Middeldorp and Hol, 2011) and is upregulated in reactive astrocytes (astrogliosis) in essentially any CNS pathology (Eng and Ghirnikar, 1994; Eng et al., 2000; Pekny and Nilsson, 2005; Sofroniew, 2009; Sosunov et al., 2013). During development, GFAP is expressed widely in a number of progenitor cell types giving rise to both neurons and glia. For example, GFAP-expressing radial glia in the ventricular zone (VZ) give rise to mature astrocytes, oligodendrocytes and neurons, as well as guiding subsequent migration of neurons (Gotz et al., 2002; Malatesta et al., 2003; Anthony et al., 2004; Merkle et al., 2004).

The seminal studies reporting the findings obtained with the GFAP KO mouse models were made by targeted deletion of the GFAP gene in ES cells (Pekny et al., 1995; Liedtke et al., 1996; McCall et al., 1996) or by targeted disruption of the GFAP gene by insertion of a *LacZ* cassette (Gomi et al., 1995). These GFAP KO mouse lines have been used to investigate whether changes in astrocyte processes and their morphological structure can influence brain morphogenesis and function, the physiology of adjacent synapses, and functional recovery after CNS insults. The overall appearance of the GFAP KO mice is indistinguishable from wild-type mice; they develop normally and display no gross alterations in behavior or CNS morphology. This observation suggests at first that GFAP is not essential for normal brain morphogenesis and function. However, closer analyses have indicated the involvement of GFAP in a wide variety of processes. First, these mice display enhanced hippocampal long-term potentiation (LTP) and deficient cerebellar long-term depression (LTD) in acute brain slices from adult mice, suggesting that GFAP intermediate filament protein is important for astrocyte–neuronal interactions and that astrocyte processes play a vital role in modulating synaptic efficacy in the CNS (McCall et al., 1996; Shibuki et al., 1996). In agreement with the cerebellar *ex vivo* findings, a significant impairment of eye blink conditioning was found in the GFAP KO mice, suggesting that GFAP is required for normal communication between Bergmann glia (specialized astrocytes) and Purkinje cells during induction and maintenance of cerebellar LTD *in vivo* (Shibuki et al., 1996). Second, morphological and functional alterations in the blood–brain barrier (BBB), disorganization of white matter architecture and vascularization, as well as hydrocephalus were reported in 18–24 month GFAP KO mice, suggesting an involvement of GFAP in the long-term maintenance of normal BBB and CNS myelination (Liedtke et al., 1996). Third, when challenged by diverse brain injuries *in vivo*, the GFAP KO mice were more vulnerable to: (i) CNS mechanical trauma (Nawashiro et al., 1998; Otani et al., 2006), (ii) cerebral ischemia (Nawashiro et al., 2000; Tanaka et al., 2002), (iii) kainic acid-induced neurotoxicity (Otani et al., 2006), and (iv) autoimmune encephalomyelitis (Liedtke et al., 1998). This was indicated by: (i) increased brain hemorrhage and mortality of mice; (ii) larger cortical infarct volume and profound decrease in cerebral blood flow; (iii) neurodegeneration; and (iv) enhanced clinical course of autoimmune encephalomyelitis and lesions, respectively. Conversely, other investigators have found that suppressing astrocytic GFAP expression in reactive astrocytes increases their basal levels of glial cell derived neurotrophic factor (GDNF), leading to improvement in neuronal survival from metabolic and excitotoxic insults (Hanbury et al., 2003). Beneficial neuroprotective and regenerative effects have also been reported after hippocampal and spinal cord injuries in mice lacking both GFAP and vimentin, another astrocyte intermediate filament protein (Kinouchi et al., 2003; Menet et al., 2003; Wilhelmsson et al., 2004). Collectively, these findings suggest that the GFAP component of the astrocyte cytoskeleton plays an important role in the physiology and pathology of the nervous system. However, because GFAP is expressed in progenitor cells during development giving rise to neurons, oligodendrocytes and astrocytes, these findings have to be viewed carefully. Inducible GFAP KO strategies can be used to circumvent this problem by removing GFAP only from astrocytes after this developmental window has closed (see below).

In light of more recent evidence suggesting that astrogliosis is not a uniform process but rather a multifaceted response with context-dependent reactions (reviewed in Sofroniew, 2009; Sofroniew and Vinters, 2010), the GFAP KO mouse models will continue to be valuable tools in future studies seeking to further unravel the function of GFAP and astrocytes in the variety of human brain challenges or diseases *in vivo*. These mouse models will assist in understanding the stages when astrocytes are engaged in beneficial or detrimental functions. Furthermore, a number of considerations can be taken into account which may lead to both a re-evaluation of earlier findings using these mice as well as open up new research directions. These include the following: (i) the heterogeneity of astrocyte morphology and physiology (Aley et al., 2006; Emsley and Macklis, 2006; Lee et al., 2006; Matyash and Kettenmann, 2010; Zhang and Barres, 2010; Oberheim et al., 2012; Freeman and Rowitch, 2013; Schreiner et al., 2013); (ii) the variability of GFAP expression levels among this heterogeneous cell population (Bernal and Peterson, 2011; Daniel-Christoph et al., 2013); (iii) the fluctuation of GFAP expression during circadian light–dark cycles, hormonal cycles, developmental, or pathological stages (Hajos, 2008); (iv) the existence of about eight alternatively spliced GFAP isoforms that may execute distinct functions in specific subsets of astrocytes (Middeldorp and Hol, 2011); and (v) the post-translational modification of these different isoforms such as phosphorylation and glycosylation that may influence GFAP assembly into intermediate filaments (Middeldorp and Hol, 2011). Suitable GFAP isoform-specific KO models will be required in future studies to address these issues. One way to investigate the impact of alternative splicing of the GFAP gene in a mouse model would be to selectively delete a single isoform. This can be accomplished by deleting a coding exon from the genome, introducing a stop codon, inactivating the splice sites responsible for generating a specific isoform, or overexpressing a dominant negative version of a certain isoform (Moroy and Heyd, 2007). An alternative way to investigate the impact of alternative GFAP splicing would be to use antisense oligonucleotides to prevent the inclusion of a particular exon in the mature mRNA (Gebski et al., 2003; Dori et al., 2005). Isoform-specific KO mouse models provide a compelling approach to study how alternative splicing of the GFAP gene may contribute to the regulation of pathophysiological CNS processes *in vivo*.

#### Role of IP_3_R2 in normal synaptic transmission and plasticity

Another global KO of a gene in astrocytes is the gene encoding the inositol-1,4,5-trisphosphate type 2 receptor (IP_3_R2). Two constitutive IP_3_R2-deficient mouse models have been obtained through a variation of the gene targeting strategy. In one, the targeting construct was generated by flanking exon 3 of IP_3_R2 with two *LoxP* sites and flanking the neo-cassette by FRT sites (Li et al., 2005). In the other, exon 1 of the gene was fused with a *LacZ* cassette (Futatsugi et al., 2005). Deletion of the IP_3_R2 subtype is a valuable tool in astrocyte research, as it is the only functional IP_3_R subtype expressed by astrocytes and the main mechanism by which astrocytes elevate intracellular Ca^2+^ levels (Petravicz et al., 2008; Agulhon et al., 2010; Di Castro et al., 2011; Takata et al., 2011). This mouse line has been critical to address the concept of gliotransmission, which stipulates that neuroactive molecules including neurotransmitters (called gliotransmitters) are released from mature passive astrocytes in a neuronally-induced and Ca^2+^-dependent manner downstream of G_q_ protein-coupled receptors (G_q_ GPCRs) to quickly modulate synaptic transmission and plasticity (illustrated and comprehensively discussed in Agulhon et al., 2008, 2012; Hamilton and Attwell, 2010). Upon G_q_ GPCR activation, IP_3_ is produced intracellularly, leading to astrocytic IP_3_R2 activation and Ca^2+^ release from the endoplasmic reticulum. *Ex vivo* and *in vivo* studies demonstrate that Ca^2+^ transients in mature passive astrocytes are driven by metabotropic G_q_ GPCRs, which can be activated by the spillover of neurotransmitters released from presynaptic terminals (Porter and McCarthy, 1996; Wang et al., 2006). Therefore, astrocytic G_q_ GPCRs are considered to be the physiological link between neuronal activity and detectable Ca^2+^ increases in mature astrocytes. However, it is important to keep in mind that future studies using improved imaging methods may reveal alternate sources of activity-driven Ca^2+^ transients in astrocytes. For example, new membrane-tethered genetically encoded Ca^2+^ indicators have allowed for detection of previously unreported constitutive Ca^2+^ transients (Shigetomi et al., 2013b; Tong et al., 2013).

The use of the IP_3_R2 KO mouse model was done to selectively obliterate the endogenous G_q_ GPCR/IP_3_R2-mediated Ca^2+^ elevations. The initial studies using this mouse model reported that removing astrocytic G_q_ GPCR/IP_3_R2-mediated Ca^2+^ fluxes did not affect basal and evoked excitatory synaptic transmission (EPSCs), or short- and long-term plasticity (NMDA receptor-dependent LTP) in the hippocampus *ex vivo* (Fiacco et al., 2007; Petravicz et al., 2008; Agulhon et al., 2010). The implications of these findings have already been well documented and discussed (Agulhon et al., 2008, 2010, 2012; Petravicz et al., 2008; Fiacco et al., 2009a; Hamilton and Attwell, 2010; Kirchhoff, 2010; Nedergaard and Verkhratsky, 2012). Recently, an *ex vivo* study replicated some of the above-described data by showing that hippocampal NMDA receptor-mediated LTP is normal in IP_3_R2 KO mice, supporting the initial findings that the IP_3_R2-mediated internal Ca^2+^ store pathway is not involved in activity-evoked gliotransmitter release at the hippocampal CA1–CA3 synapses (Shigetomi et al., 2013b). Moreover, the authors of this study discovered a novel mechanism based on transient receptor potential A1 (TRPA1)-mediated transmembrane Ca^2+^ fluxes, through which astrocytes can modulate LTP. They found that pharmacological blockade or genetic deletion of a recently described TRPA1 channel in astrocytes alters free basal Ca^2+^ levels, leading to a decrease in Ca^2+^-dependent constitutive/homeostatic release of D-serine, and thus LTP reduction – in support of a previous study (Henneberger et al., 2010). Such Ca^2+^ rises are not the type of neuronally-induced astrocyte Ca^2+^ elevations suggested to drive fast release of gliotransmitters by mature astrocytes. Overall these findings emphasize how different astrocyte Ca^2+^ sources (i.e., activity-evoked G_q_ GPCR/IP_3_R2-dependent Ca^2+^ elevations vs*.* constitutive TRPA1-dependent basal Ca^2+^ dynamics) have distinct effects on LTP (Shigetomi et al., 2013b), and potentially other physiological effects previously attributed to IP_3_R2-driven astrocyte Ca^2+^ elevations.

Recent studies using IP_3_R2 KO mice *in vivo* reported that hippocampal muscarinic or cortical NMDA receptor-mediated LTP is diminished or abolished, suggesting a role for IP_3_R2-driven Ca^2+^ elevations in synaptic plasticity (Takata et al., 2011; Navarrete et al., 2012). Two exciting new reports are providing a potential explanation for the differences observed between the early and later studies using the IP_3_R2 KO. By using a broad range of G_q_GPCR antagonists, Wang and coauthors excluded the occurrence of gliotransmission both *ex vivo* and *in vivo* (Wang et al., 2012a,b), consistent with the prior *ex vivo* studies using the IP_3_R2 KO mice. Rather, they identified a mechanism by which astrocytic G_q_ GPCR/IP_3_R2-mediated Ca^2+^ elevations stimulate the Na^+^, K^+^-ATPase, leading to a transient K^+^ uptake by astrocytes, a decrease in the extracellular K^+^ concentration, and a subsequent modulation of excitatory postsynaptic currents. This modulation of synaptic transmission was not observed in the IP_3_R2 KO mice, implying that astrocyte G_q_ GPCR/IP_3_R2/Ca^2+^-induced decrease of extracellular K^+^ concentration endows astrocytes with a simple and powerful mechanism for rapid modulation of neuronal activity (Wang et al., 2012a,b). Modulation of K^+^ uptake by astrocytic G_q_ GPCRs was also observed *ex vivo* (Devaraju et al., 2013; Wang et al., 2013). Rapid modification of K^+^ uptake provides a mechanism which may be responsible for effects on neuronal activity that hitherto have been ascribed to gliotransmission. Nevertheless, synaptic properties are variable within the nervous system, and astrocytes represent a genetically and functionally heterogeneous group of cells (Zhang and Barres, 2010; Oberheim et al., 2012); these cells are likely to exhibit different functions in different areas of the CNS or even within the same area. Therefore, the different effects of knocking out IP_3_R2 on neuronal activity support the need to consider heterogeneity of astrocytes between different brain regions when comparing data across studies. Further investigation is necessary to determine the mechanisms involved in activity-induced astrocytic G_q_ GPCR/IP_3_R2/Ca^2+^-mediated modulation of neuronal excitability and LTP *in vivo*.

In our view, a fundamental and still open question remains to be answered in order to address this issue thoroughly: do neurons express IP_3_R2? If so, does this neuronal receptor subtype play a role in specific types of synaptic transmission and plasticity? Fully deleting IP_3_R2 abolishes spontaneous and activity- or agonist-dependent G_q_ GPCR/IP_3_R2-mediated Ca^2+^ increases in astrocytes but leaves intact neuronal Ca^2+^ signaling (Petravicz et al., 2008; Di Castro et al., 2011; Takata et al., 2011; Chen et al., 2012; Navarrete et al., 2012; Wang et al., 2012a,b). However, caution should be exercised in the interpretation of the positive physiological data using this mouse model. Indeed, past immunohistochemical studies aimed at identifying the expression of IP_3_R2 in neurons were inconclusive (Sharp et al., 1999; Holtzclaw et al., 2002; Hertle and Yeckel, 2007), and most recent antibodies against this IP_3_R subtype are of poor quality (Takata et al., 2011; Chen et al., 2012). Additionally, functionally testing whether neurons display IP_3_R2-mediated Ca^2+^ signaling would require the use of double KOs for the two other IP_3_R subtypes (IP_3_R1 and IP_3_R3), which is complicated because most of the IP_3_R1-deficient mice die *in utero* (Matsumoto et al., 1996). We are then left with the possibility that some neurons, which also have heterogeneous subtypes throughout the brain, may express IP_3_R2 in addition to IP_3_R1 and/or IP_3_R3. Such a hypothesis, if validated, could help address some of the discrepancies between positive and negative findings using the IP_3_R2 KO mice. The use of inducible conditional IP_3_R2 KO mice to selectively knock out IP_3_R2 in neurons vs*.* astrocytes in adult mice appears to be the next obvious step to help differentiate between neuronal vs*.* astrocytic G_q_ GPCR effects on neuronal excitability and LTP *in vivo*. Finally, the currently available and future inducible IP_3_R2 KO mouse models may help provide insight into brain pathology; recent studies suggest that abnormal (increased) astrocytic Ca^2+^ dynamics are linked to the symptoms of Alzheimer’s disease (Takano et al., 2007; Kuchibhotla et al., 2009; Grolla et al., 2013), suggesting that controlling astrocytic Ca^2+^ homeostasis might be a potential form of therapy for neurodegenerative disease.

### CONDITIONAL AND INDUCIBLE CONDITIONAL KNOCKOUT MOUSE MODELS

**FIGURE 2:**
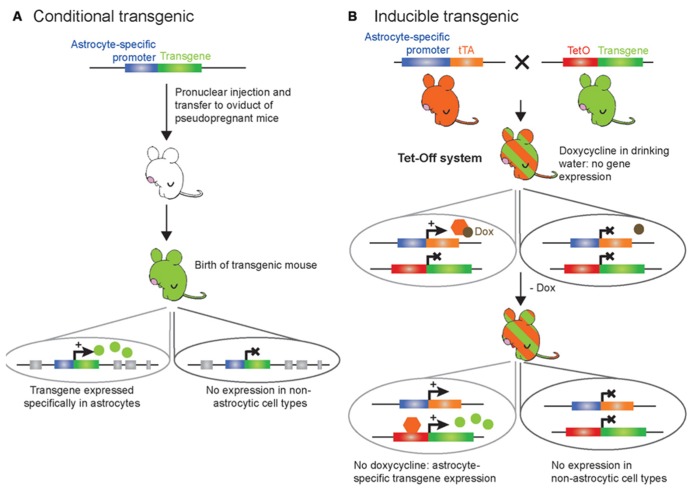
**Schematic representation of the genetic modification systems to make astrocyte-specific transgenic mice.** **(A)** Conventional strategy for conditional expression of a transgene selectively in astrocytes. A transgene of interest is placed under the control of an astrocyte-specific promoter; this cDNA construct is purified and microinjected into the male pronucleus of fertilized murine eggs. Several copies of the DNA will be randomly inserted in the mouse genome. Then, the eggs are transferred to the oviduct of pseudopregnant females. The resulting offspring (green) deriving from the eggs will display constitutive transgene expression selectively in astrocytes as soon as the astrocyte-specific promoter is active, while it will not be expressed in other cell types. **(B)** Tet-Off strategy for inducible and temporal control of transgene expression selectively in astrocytes. The TeT-Off system has been used more frequently than the Tet-On system, and is based on the tetracycline (tet) bacterial resistance gene operon. The creation of two transgenic lines is required. The first transgenic line (orange) contains the tTA under the control of an astrocyte-specific promoter: this line provides astrocyte selectivity to the system. The second transgenic line (green) contains the transgene of interest under the control of the tet operon (TetO) DNA-binding element fused to a minimal promoter from cytomegalovirus. The TetO promoter element can be activated upon binding of the tetracycline transactivator (tTA) to drive ubiquitous expression. When a tetracycline derivative, such as doxycycline, is given to the bigenic mice (green/orange-striped – obtained from breeding the tTA line with the TetO line) in the drinking water, doxycycline binds to tTA to prevent its interaction with the TetO minimal promoter and thereby turn off transgene expression selectively in astrocytes. Removing doxycycline from the drinking water of the bigenic mice leads to binding of tTA to the TetO minimal promoter and transcription of the transgene of interest within a week. This allows desired temporal expression of the transgene selectively in astrocytes while it is not expressed in other cell types. Additionally, this system allows testing whether potential physiological or behavioral effects of the transgene are reversible by putting the bigenic mice back on doxycycline.

The complete knock out of genes in mice has allowed researchers to investigate the role of specific genes *in vivo*. When a gene is selectively expressed in a specific cell type and/or tissue, a constitutive KO is thus cell-specific and/or tissue-specific. However, it is rare that genes are expressed by a single cell type. In such instances, the phenotypes of KO mice can be very complex to interpret and it is not uncommon for a KO mouse to display embryonic lethality (Matsumoto et al., 1996), show no phenotype at all, or affect other gene products (Iacobas et al., 2004). To overcome these obstacles, genes have been knocked out in a cell-specific manner with the use of Cre recombinase/*LoxP* technology. Such gene KOs are referred to as conditional gene KOs (cKO; **Figure 1B**). The site-specific recombination system of the P1 bacteriophage using Cre and *LoxP*-flanked genes is well documented (Sternberg and Hamilton, 1981), although it was only much later that it was used in mouse lines to induce cell-specific gene cKOs in non-nervous and/or nervous tissues (Gu et al., 1994; Morozov et al., 2003). In order to accomplish this, two mouse lines are required: one that has been genetically engineered to express the Cre site-specific DNA recombinase of bacteriophage P1 under a cell-specific promoter (transgenic mice; **Figure 2A**), and a second that has been made via homologous recombination in ES cells expressing the 34-base-pair *LoxP* site (recognition site for Cre recombinase) flanking the gene of interest or one or more exons of this gene. When these two mouse lines are crossed, the gene or exons of interest are excised to obtain the desired cKO mouse line (reviewed in Sauer, 1998; **Figure 1B**). While cKO mice are very valuable tools, their use has been limited in the astrocyte field because the promoters that are known to be astrocyte specific in the adult are also expressed in progenitor cells of the developing brain. As a consequence, not only astrocytes, but also a large percentage of neurons and oligodendrocytes, exhibit recombination when using astrocyte-specific promoters to drive the expression of Cre recombinase (Gotz et al., 2002; Malatesta et al., 2003; Anthony et al., 2004; Merkle et al., 2004; Casper and McCarthy, 2006). This limitation prompted investigators in the field to develop transgenic mice that enable *inducible* cell-specific gene KOs in order to recombine astrocytic genes postdevelopmentally (referred to as inducible cKO mice; **Figure 1C**). To this end, mice have been developed which express Cre recombinase fused to a mutated form of the human estrogen receptor (ER^T2^) that restricts the fusion protein to the cytoplasm unless exposed to the estrogen analog tamoxifen (or 4-hydroxy-tamoxifen; Feil et al., 1997). This form of the Cre recombinase, called CreER^T2^, enters the nucleus to cause cell-specific recombination only when tamoxifen is given to mice (Hayashi and McMahon, 2002). Thus, genetically engineered mice expressing CreER^T2^ under the control of an astrocyte-specific promoter (e.g., GFAP, glutamate transporter GLAST, Aldh1l1 or Cx43) allows study of the effect of gene deletion specifically in astrocytes if tamoxifen is given to mice at later development stages (Eckardt et al., 2004; Hirrlinger et al., 2006; Mori et al., 2006; Casper et al., 2007; **Figure 1C**). One limitation of the CreER^T2^ inducible system and inducible systems in general is that recombination efficiency may be low and therefore the phenotypes generated may be more subtle, as has been reported for Cx43 compared to floxed reporter genes (Casper et al., 2007).

#### Role of CB_1_R in working memory

Multiple inducible cKO mice have been recently developed to study astrocytic function *in vivo*. One example is a tamoxifen-inducible cKO mouse specifically lacking cannabinoid type-1 receptor (CB_1_R) expression in astrocytes (Han et al., 2012). A mouse line carrying a *LoxP*-flanked CB_1_R (Marsicano et al., 2003) was crossed with a line expressing CreER^T2^ under the control of the human GFAP promoter (Hirrlinger et al., 2006). The resulting new line of mice (GFAP-CB_1_R-KO) has been used to investigate the mechanisms underlying impairment of working memory, which is one of the most important deleterious effects of marijuana intoxication in humans (Ranganathan and D’Souza, 2006) and animals (Lichtman and Martin, 1996; Wise et al., 2009). Understanding the side-effects associated with the use of marijuana has important clinical implications because the derivatives of marijuana or synthetic cannabinoids represent promising therapeutic molecules for several human conditions, including pain, nausea, seizures, ischemia, cerebral trauma, and tumors (Lemberger, 1980; Carlini, 2004; Hall et al., 2005). The endocannabinoid system has recently emerged as an important neuromodulatory system, and the CB_1_R (GPCR predominantly coupling to G_i_ proteins) is highly abundant in the CNS (Herkenham et al., 1990; Matsuda et al., 1990). In particular, the CB_1_R is expressed in glutamatergic and GABAergic neurons (Herkenham et al., 1990; Kawamura et al., 2006) as well as in astrocytes (Navarrete and Araque, 2008; Han et al., 2012) of the hippocampal CA1 area, an area that contributes to spatial working memory (SWM). Because past studies have established that CB_1_Rs mediate retrograde inhibition of neurotransmitter release, control neuronal excitability, and regulate short- and long-term plasticity (Di Marzo et al., 1994; Kreitzer and Regehr, 2001; Alger, 2002; Wilson and Nicoll, 2002; Freund et al., 2003; Chevaleyre et al., 2006; Maldonado et al., 2006; Heifets and Castillo, 2009; Kano et al., 2009), research on the function of CB_1_R signaling in pain (Kato et al., 2012), aversive memory (Marsicano et al., 2002), epilepsy (Monory et al., 2006), food intake (Bellocchio et al., 2010), analgesia (Zimmer et al., 1999), or development (Jin et al., 2004) has focused mainly on neuronal CB_1_Rs using neuronal-specific CB_1_R KO mouse models. However, Han et al. (2012) report an unappreciated, yet major, role of astrocytic CB_1_Rs in SWM. Their results show that acute cannabinoid exposure *in vivo* elicits a previously unreported form of LTD at CA3–CA1 hippocampal synapses, which is associated with an impairment of SWM; both effects are abolished in tamoxifen-treated GFAP-CB_1_R-KO, but conserved in mice lacking CB_1_R in glutamatergic or GABAergic neurons (Han et al., 2012). These findings strongly suggest that astrocytic CB_1_R is a primary mediator of cannabinoid-induced LTD. Based on further *in vivo* pharmacological studies, the authors speculated that their findings are consistent with the possibility that acute exogenous cannabinoid exposure leads to glutamate release from astrocytes, which in turn could activate extrasynaptic NR2B-containing NMDA receptors to trigger AMPA receptor internalization at CA3–CA1 synapses. These events could eventually induce cannabinoid-mediated LTD at these synapses, altering hippocampal SWM. However, several points should to be kept in mind when considering this interpretation of the data, which might imply a substantially more complex mechanism at the CA1–CA3 synapses: (i) the apparent mechanistic discrepancy between this *in vivo* cannabinoid-induced LTD and previous *ex vivo* “gliotransmission” studies suggesting that astrocytic CB_1_R-mediated Ca^2+^ increases trigger the release of glutamate to activate pre-synaptic mGluRs that leads to glutamate presynaptic release and subsequent potentiation of postsynaptic NMDA receptor-mediated currents (Navarrete and Araque, 2008, 2010); (ii) the current debate as to whether astrocytes actually release glutamate in a GPCR/Ca^2+^-dependent manner *in vivo* (Wang et al., 2012a compared to Navarrete et al., 2012); (iii) the emerging evidence that astrocyte GPCR/Ca^2+^-dependent release of glutamate occurs in the early steps of inflammatory processes rather than during normal physiology (Agulhon et al., 2012; Pascual et al., 2012); (iv) the recent findings suggesting that astrocytic GPCR/Ca^2+^ triggers an increase of K^+^ uptake (Wang et al., 2012a,b; Devaraju et al., 2013); and (v) a past study reporting that LTD is modulated by astrocytic K^+^ uptake (Janigro et al., 1997). Further investigation should help determine whether cannabinoid-induced LTD is due to an increase of extracellular glutamate, a decrease of extracellular K^+^*,* a combination of both, or some other mechanism.

#### Role of DRD2 in neuroinflammation

Astrocytes become reactive in nearly all brain pathologies, and play important roles in neuroinflammation, a common feature of the aging brain and most neurological disorders and neurodegenerative diseases (Sofroniew, 2009; Sofroniew and Vinters, 2010). Changes in reactive astrocytes include upregulation of GFAP expression (Pekny and Nilsson, 2005), secretion of inflammatory mediators from astrocytes (Lucas et al., 2006; Peng et al., 2006; Farina et al., 2007; Brambilla et al., 2009; Steele and Robinson, 2012), and altered expression of astrocytic GPCRs (Aronica et al., 2003; Hamby et al., 2012). One such GPCR is the dopamine D2 receptor (DRD2) that couples to G_i_ (Missale et al., 1998) and is expressed not only in neurons, but also in astrocytes (Bal et al., 1994; Khan et al., 2001; Luo et al., 2009). Downregulation of DRD2 expression has been reported in the brain of the elderly (Kaasinen et al., 2000), suggesting that astrocytic DRD2 signaling may be involved in neuroinflammation that occurs in the CNS during aging and disease. A recent study has investigated this question using both astrocyte-specific cKO and tamoxifen-inducible cKO mouse models (Shao et al., 2013). Several complementary methodological approaches have been used, including immunohistochemistry, biochemistry, and molecular biology to show that DRD2-deficient astrocytes show robust GFAP upregulation in the substantia nigra of aged mice. Moreover, DRD2-deficient astrocytes were found to produce more proinflammatory mediators than their wild-type counterparts, suggesting that astrocytic DRD2 is a key negative regulator of neuroinflammation. To identify the downstream effectors of DRD2 that might be involved in the regulation of inflammatory mediator production, microarray analysis was carried out and showed a pronounced decrease of the small heat-shock protein αB-crystallin (CRYAB) in DRD2-deficient astrocytes, a protein known to display anti-inflammatory and neuroprotective activities (Ousman et al., 2007; Bhat and Steinman, 2009). These findings suggest that activation of astrocytic DRD2 controls CRYAB expression to suppress inflammatory responses in astrocytes and thus contribute to the maintenance of the immune state balance under normal conditions (Shao et al., 2013). The implications of these findings are profound in age-related diseases, where a decrease of DRD2 signaling in astrocytes may lead to an abnormal increase of proinflammatory mediator release, and underlie the progression of cognitive and motor function impairments. Therefore, the astrocyte DRD2 signaling pathway could be a potential target for therapy of several neurological, neuroinflammatory, and neurodegenerative disorders, in which production of inflammatory mediators is a common element (Lucas et al., 2006).

It is interesting, however, to note that activation of another G_i_ GPCR, called CXCR4 in cultured cortical astrocytes has previously been reported to increase release of the proinflammatory cytokine tumor necrosis factor α (TNFα; Bezzi et al., 2001), and not to negatively control the release of proinflammatory molecules as reported by Shao et al. (2013). Several obvious possibilities may explain these differences: (i) astrocytic G_i_ signaling triggers different cellular mechanisms depending on whether the studies are performed *in vivo* vs*.* *ex vivo* cultures; (ii) astrocytic G_i_ signaling leads to distinct responses depending on the area of the brain (e.g., substantia nigra vs*.* cortex); or (iii) astrocytic G_i_ GPCRs are involved in different functions depending on the developmental stage (mature vs*.* immature astrocytes). Additionally, another possibility that has not been addressed thoroughly in the field when investigating the role of astrocyte signaling in general is that different astrocytic G_i_ GPCRs may activate distinct signaling pathways even though they are known to couple to G_i_ proteins. In other words, depending on the endogenous or synthetic agonist used to active a specific G_i_ GPCR, this receptor may show a different bias toward other G proteins over G_i_ or different functional selectivity for β-arrestin recruitment over G_i_ activation (Allen et al., 2011; Zidar, 2011; Audet et al., 2012; Blattermann et al., 2012; Hiller et al., 2013). The depth of the complexity of GPCR signaling is now becoming familiar territory to receptor biologists, yet the application of this knowledge to the astrocyte field remains extremely limited. Therefore, findings relative to astrocytic GPCR signaling (coupling to G_q_, G_i_, or G_s_ proteins) in physiology and pathology likely reflect the complex interactions of multiple signaling pathways and provides an explanation for seemingly contradictory observations. Examples of this have already been observed relative to astrocyte Ca^2+^ signaling which is traditionally ascribed to G_q_ GPCR signaling pathways, yet astrocytic G_i_-coupled GABA_B_ and CB_1_Rs also have been reported to evoke Ca^2+^ elevations in astrocytes (Serrano et al., 2006; Navarrete and Araque, 2008). Future studies using biased ligands in combination with astrocyte-specific cKOs of specific G proteins or β-arrestins is one approach to elucidate the role of astrocyte signaling molecules in brain function and dysfunction.

## STUDYING ASTROCYTE FUNCTION THROUGH GENE EXPRESSION OR CELL-SPECIFIC RESCUE OF GLOBAL GENE KNOCKOUT

As methods for generating full or inducible conditional gene KO have been useful in deciphering some of the astrocyte gene functions *in vivo*, constitutive, inducible, and/or reversible regulation of gene expression represent other powerful approaches for understanding astrocyte function. To this end, four molecular approaches can be used: (i) expression of a genetically modified gene (transgene); (ii) reversible regulation of an endogenous gene; (iii) viral transduction of genes; or (iv) *in utero* gene electroporation (IUE).

### TRANSGENE EXPRESSION

Conventional (**Figure 2A**) and inducible (**Figure 2B**) transgenesis allowing for astrocyte-specific expression of transgenes, constitutively or at specific times, have been used in the field. These approaches employ a small transcriptional unit derived from astrocyte-specific promoters. Of importance for the topic of this review, mapping of the transcriptional regulatory elements of the GFAP promoter has been critical to develop regulatory units (promoters) of small size to direct transgene expression in the majority of astrocytes *in vivo* without significant expression in other cell types in the brain (Masood et al., 1993; Brenner, 1994; Brenner et al., 1994). Other astrocyte-specific promoters have also been used successfully to drive transgene expression in astrocytes, such as Cx30, Cx43, S100b, Aldh1l1, or GLAST promoters (reviewed in Pfrieger and Slezak, 2012). The DNA construct containing a promoter fused to the transgene of interest is microinjected into the male pronucleus of fertilized eggs for random insertion in the mouse genome, followed by transfer of the fertilized eggs to the oviduct of pseudopregnant recipient females (Cho et al., 2009; **Figure 2A**). The resulting pups are identified as transgenic by polymerase chain reaction and checked for astrocyte-specific transgene expression. This constitutive gain-of-function approach however does not provide temporal control of the transgene expression, which can be problematic when studying the function of a transgene during developmental stages as mentioned above (Gotz et al., 2002; Malatesta et al., 2003; Anthony et al., 2004; Merkle et al., 2004; Casper and McCarthy, 2006). To overcome this limitation, inducible transgenic mouse models have been developed. The most commonly used method to temporally control gene expression in mouse models is based on the tet-operon/repressor and the estrogen (tetracycline) receptor ligand-binding domain (Gossen and Bujard, 1992; Baron and Bujard, 2000; Mansuy and Bujard, 2000; Saunders, 2011; **Figure 2B**). This system is bi-transgenic, which means that it involves the mating of two different transgenic mouse lines in order to produce a new tetracycline-regulated transgenic mouse line designed to activate the expression of a transgene in a specific cell type at a specific time point. The first line contains an astrocyte-specific promoter driving the expression of the tetracycline (tet) transactivator (tTA). The second line carries the transgene of interest driven by the tet operon (TetO) DNA-binding element fused to a minimal promoter from cytomegalovirus. In this system, bigenic mice are maintained on tetracycline (doxycycline) to block transgene expression. When doxycycline is removed at a given time, tTA then binds to the TetO minimal promoter leading to targeted expression of the transgene of interest (**Figure 2B**).

#### Role of astrocytic G_q_ GPCR signaling in physiology and behavior

One important limitation in addressing the role of astrocytic GPCR signaling in neurophysiology has been the inability to pharmacologically activate astrocytic GPCRs in a cell type-specific manner. Indeed, astrocytes *in vivo* express members of most of the different families of GPCRs linked to the diverse array of intracellular signaling cascades (Porter and McCarthy, 1997) that are also known to be expressed by neurons. Therefore, the use of pharmacological approaches consisting of agonist application *ex vivo* or *in vivo* does not allow cell-specific GPCR activation. Therefore, interpretation of the findings is made difficult by direct activation of neuronal (but also other cell type) receptors by the applied agonist in addition to the intended astrocytic targets. As a consequence, it has been difficult to determine the effect of selectively stimulating astrocytic G_q_ GPCR-mediated Ca^2+^ signaling cascades on physiological processes such as synaptic transmission and plasticity. This complication has led investigators to consider previous reports of gliotransmission with caution.

Thus, advances in this field depend upon the development of novel tools to better address the physiological relevance of astrocytic G_q_ GPCR Ca^2+^ signaling. In order to overcome some of the limitations associated with traditional pharmacological approaches, two novel transgenic mouse models were developed (Fiacco et al., 2007; Agulhon et al., 2013). In the first model, a novel G_q_ GPCR is expressed selectively in astrocytes that is not expressed by other cell types in the brain, is not activated by endogenous ligands released in brain, and whose ligand, the peptide FMRF, does not activate endogenous brain G_q_ GPCRs (Fiacco et al., 2007). The novel receptor, the so-called Mas-related gene A1 (MrgA1), is a member of a family of GPCRs normally expressed in specific subsets of nociceptive sensory neurons in the spinal cord (Dong et al., 2001), but is not found in the brain. The MrgA1 receptor is targeted to astrocytes using the inducible Tet-Off system (**Figure 2B**). In one transgenic mouse line the green fluorescent protein (GFP)-tagged MrgA1 receptor is transcribed from the TetO promoter. When crossed with a second transgenic line in which tTA is targeted to astrocytes using the human GFAP promoter, a bigenic line (referred to as the MrgA1 mice) is obtained, in which MgrA1-GFP is selectively expressed in the vast majority of astrocytes in the absence of doxycycline (Fiacco et al., 2007). Several studies using this novel transgenic MrgA1 mouse model have shown that increasing astrocytic MrgA1-mediated Ca^2+^ fluxes does not lead to gliotransmission *ex vivo* and *in vivo*, suggesting that astrocytes do not release gliotransmitters in a G_q_ GPCR/Ca^2+^-dependent manner (Fiacco et al., 2007; Agulhon et al., 2010; Wang et al., 2012a,b). Rather, it has been reported that astrocytic MrgA1R-mediated Ca^2+^ elevations potentiate astrocyte glutamate and K^+^ uptake (Wang et al., 2012a,b, 2013; Devaraju et al., 2013), suggesting that the mechanisms by which agonist-induced astrocyte G_q_ GPCR activation modulates neuronal activity may be different than previously thought, in agreement with previous and most recent findings (Fiacco et al., 2007; Agulhon et al., 2010; Wang et al., 2013). It is important to bear in mind that agonist-evoked stimulation of G_q_ GPCRs, including MrgA1Rs, generates Ca^2+^ elevations that may not recapitulate some endogenous Ca^2+^ elevations in astrocytes which often remain confined to fine processes. One strategy in future studies could be to employ a caged version of FMRF to locally stimulate astrocyte Ca^2+^ elevations by activation of a G_q_ signaling pathway, which may more closely resemble the subset of microdomain astrocytic Ca^2+^ elevations which have been observed in astrocytes *in vivo*.

More recently, another transgenic mouse line has been developed in order to facilitate investigation of the role of astrocytic G_q_ GPCR signaling *in vivo* (Agulhon et al., 2013). One of the limitations of the MrgA1 mice is that the MrgA1 FMRF ligand does not cross the BBB, making *in vivo* studies more invasive as surgeries would be required in order to infuse FMRF into the brain of freely moving mice. Additionally, endogenous MrgA1 is expressed in sensory nerve terminals, preventing the use of MrgA1 mice to study spinal cord astrocytes (as FMRF may diffuse and activate endogenous MrgA1 in nociceptive sensory neurons). To overcome some of these obstacles, a new GFAP-hM3Dq mouse line was created (Agulhon et al., 2013). The innovation of this mouse line is based on the use of a novel genetically engineered G_q_ GPCR (called hM3Dq) that does not respond to endogenous ligands, but instead responds to an inert synthetic ligand (clozapine-*N*-oxide, CNO) that crosses the BBB and activates signaling cascades in a similar fashion as endogenous G_q_ GPCRs (Armbruster et al., 2007). Such new chemogenetic technology (called designer receptors exclusively activated by a designer drug, or DREADD) has recently led to several important discoveries in neuronal function (e.g., Alexander et al., 2009; Ferguson et al., 2011; Atasoy et al., 2012; Garner et al., 2012), and is now being applied to astrocytes for the first time (Agulhon et al., 2013). This mouse line was made through conventional transgenesis using a construct containing the human GFAP promoter driving the expression of hM3Dq (**Figure 2A**). Inducible and reversible expression of the transgene is not a constraint when using the DREADD technology, as hM3Dq can be activated postdevelopmentally through intraperitoneal CNO injection. Immunohistochemical screening in adult mice indicates that expression of hM3Dq is restricted to astrocytes within the CNS, and in non-myelinating Schwann cells of sympathetic, sensory, and sciatic nerves, as well as satellite cells in sympathethic, parasympathetic, and sensory ganglia within the PNS. Such expression is expected for a GFAP-driven transgene and allows CNO-induced global stimulation in GFAP^+^ glial cells within the CNS and PNS. This provides a powerful approach, as it can reveal most of the physiological and behavioral phenotypes mediated by GFAP^+^ glial cell G_q_ GPCR signaling. Acute CNO intraperitoneal injections of GFAP-hM3Dq mice resulted in previously unreported and long-lasting (minutes to hours) modulation of autonomic nervous system (ANS) function, including increased heart rate, blood pressure, and saliva formation, as well as decreased body temperature. Furthermore, changes in activity-related behavior and motor coordination were observed in CNO-treated GFAP-hM3Dq mice. To address whether ANS and activity-related effects were due to Ca^2+^ or other signaling pathways downstream of G_q_ GPCR activation, the GFAP-hM3Dq mice were crossed with IP_3_R2 KO mice in order to generate GFAP-hM3Dq mice in which IP_3_R2-dependent Ca^2+^ increases were abolished. Interestingly, CNO-treated GFAP-hM3Dq/IP_3_R2-deficient mice exhibited similar autonomic and motor modulation as CNO-treated GFAP-hM3Dq mice. These findings suggest that CNO-induced phenotypes are not dependent on IP_3_R2-dependent Ca^2+^ increases. Thus, other (non-Ca^2+^) signaling molecules activated by G_q_ GPCRs in GFAP^+^ glial cells may be important contributors to the functional effects of CNO. Collectively, these findings open new avenues into investigation of GFAP^+^ glial cell (astrocyte, non-myelinating Schwann cell, satellite cell) function in animal physiology. Further studies employing local CNO infusion into specific regions of the CNS or PNS, and specific blockers of G_q_ GPCR signaling molecules (e.g., protein kinase C or βγ-dependent activation of signaling cascades), will help dissect out the areas of the nervous system and the signaling pathways that are responsible for each of the CNO-induced phenotypes. Viral transduction of hM3Dq in wild-type mice is also a potential strategy to help determine what areas of the CNS vs*.* PNS are involved in the effects observed in CNO-treated GFAP-hM3Dq mice. Finally, when specific markers of different subpopulations of CNS astrocytes become available, it will be possible to use new promoters to create transgenic mice expressing hM3Dq in distinct subsets of astrocytes. This will allow determination of whether a specific subset of astrocytes is responsible for all of the phenotypes observed in CNO-treated GFAP-hM3Dq mice, or whether different subsets of astrocytes are responsible for distinct phenotypes. Clearly, there is much to be learned with regard to the role of astrocytes, and more generally of GFAP^+^ glial cells, in complex physiology and behavior. Collectively, the findings of this study point to astrocytes as potential therapeutic targets for some ANS or motor dysfunctions.

### CELL-SPECIFIC RESCUE OF A GENE KNOCKOUT

Another powerful molecular approach is to constitutively knock out a particular gene product and then rescue it in a specific cell population. This provides information on the contribution of a specific cell type to the phenotype produced by the full KO of the protein. This technique employs a *LoxP*-flanked selectable neo marker and transcriptional/translational stop cassette (called neostop) located in the endogenous gene of interest to generate a null mutant that can be activated by Cre-mediated recombination. This construct is placed into a targeting vector, which is inserted into ES cells and introduced into the mouse genome through homologous recombination (Müller, 1999; **Figure 1A**) to eventually generate a mouse line in which expression of the targeted endogenous gene is suppressed. When this Cre responding mouse line is crossed with a transgenic line expressing Cre recombinase under a cell type-specific promoter, recombination of *LoxP* sites excises the neostop cassette, thus re-activating expression of the endogenous gene conditionally (**Figure 1B**) and with temporal control in a specific cell population (**Figure 1C**). This approach is powerful in the sense that it enables study of important questions regarding the phenotype reversibility of certain diseases involving the loss of a single gene function, and thus has the potential to open doors for future therapeutic approaches (Dragatsis and Zeitlin, 2001). By providing gain- and loss-of-function information, this approach has led to recent important steps forward in the understanding of the role of astrocytes in autism spectrum disorders.

### ROLE OF ASTROCYTE MeCP2 IN RETT’S SYNDROME

Rett’s syndrome (RTT) is an X-chromosome-linked autism spectrum disorder caused by the loss of function of the epigenetic factor methyl-CpG-binding protein 2 (MeCP2; Amir et al., 1999; Chahrour and Zoghbi, 2007). MeCP2 aberrations result in a constellation of neuropsychiatric, neuroanatomical, and neurophysiological abnormalities as well as autonomic dysfunctions, such as respiratory abnormalities. Delayed neuronal maturation and synaptogenesis, sparse and short dendritic spines (Fukuda et al., 2005), impaired synaptic transmission and plasticity (Dani et al., 2005; Asaka et al., 2006; Moretti et al., 2006), and altered number of glutamatergic synapses and expression of excitatory glutamate transporter VGLUT1 (Chao et al., 2007) were detected in global MeCP2 KO mouse lines (also called RTT mouse models). Although no unifying principle on MeCP2 function has yet emerged, it has been reported that MeCP2 acts as a transcriptional repressor, activator or RNA-binding protein (Nan et al., 1998; Colantuoni et al., 2001; Chahrour et al., 2008). Most studies have been directed toward understanding the *in vivo* mechanisms of neuronal MeCP2 (Akbarian et al., 2001) using different neuronal-specific cKO mouse models, which led to the thought that the primary cause of RTT is cell autonomous, i.e., resulting from a lack of functional MeCP2 in neurons (Chen et al., 2001; Guy et al., 2001; Luikenhuis et al., 2004). However, more recent studies have shown that MeCP2 is also expressed in all glial cell types, including astrocytes, oligodendrocyte progenitor cells, oligodendrocytes, and microglia (Ballas et al., 2009; Derecki et al., 2012). In particular, *in vitro* studies have shown that astrocytic MeCP2 supports normal neuronal morphology, indicating a non-cell autonomous influence of MeCP2 on neuronal function (Ballas et al., 2009; Maezawa et al., 2009). Global re-expression of the MeCP2 gene postnatally in full MeCP2 KO mice demonstrated disease reversibility in this RTT mouse model, suggesting that the neurological defects in MeCP2 disorders are not permanent (Guy et al., 2007). Based on these studies, Lioy et al. (2011) asked whether astrocytic MeCP2 may also have a role in rescuing RTT neuropathological symptoms *in vivo* in order to determine the contribution of astrocytes to the symptoms of RTT. They used a genetically engineered mouse line in which the endogenous MeCP2 gene is globally silenced by insertion of a *Lox*P-flanked stop cassette, but can be conditionally activated by stop cassette deletion *via* Cre-mediated excision (MeCP2^lox-Stop^ mice; Guy et al., 2007). When these MeCP2^lox-Stop^ mice were crossed with mice expressing CreER^T2^ under a human GFAP promoter (hGFAP–CreER^T2^), (Hirrlinger et al., 2006), the resulting new mouse line (MeCP2^lox-Stop^::hGFAP–CreER^T2^) was thus specifically designed for the investigation of astrocytic function in RTT (Lioy et al., 2011). The MeCP2^lox-Stop^::hGFAP–CreER^T2^ mice are globally deficient in MeCP2 until injected with tamoxifen, which induces the excision of the Lox-stop cassette in the endogenous MeCP2 gene, restoring the expression of MeCP2 in an astrocyte-specific manner. Strikingly, it was found that re-expression of MeCP2 specifically in astrocytes significantly improved locomotion, anxiety levels, breathing patterns, and average life span, indicating that astrocytes are involved in the neuropathology of RTT and that restoring astrocyte MeCP2 can ameliorate four consistent and robust RTT-like symptoms. Additionally, restoration of MeCP2 in astrocytes in the global KO mice exerted a non-cell-autonomous positive effect on KO neurons *in vivo*, restoring normal dendritic arborization and increasing levels of VGLUT1. Altogether, these findings suggest that astrocytic MeCP2 gene replacement is well-suited as a therapeutic strategy. Therefore, these findings have major implications not only for improving the understanding of astrocyte function in pathophysiology but also have valuable clinical implications (Gadalla et al., 2013; Garg et al., 2013). Deciphering the cellular (astrocyte vs*.* neuron) and molecular underpinnings of RTT is likely to contribute to the understanding of the pathogenesis of a broader class of autism spectrum disorders. Finally, the use of conditional endogenous gene repair mutations has a clear application for studying astrocytic contributions to other single gene diseases.

### VIRAL GENE TRANSDUCTION STRATEGIES

A new technique that is gaining momentum in the study of astrocyte function is viral-mediated delivery of transgenic constructs to express or perturb a protein or molecule of interest. This strategy offers an attractive alternative to generation of transgenic mouse lines, which can be very time consuming and expensive due to costs associated with maintenance of animal lines and genotyping. Viral mediated gene delivery is reviewed in detail in this special issue (Merienne et al., 2013) and therefore this section will focus mainly on a particular application of this approach for the study of astrocyte Ca^2+^ activity.

Briefly, in these experiments, a transgenic construct is inserted into a recombinant AAV vector which is then injected into the brain of wild-type mice. Approximately 2 weeks is sufficient for the viral vector to be incorporated into the host genome and for high expression of the transgenic construct to occur (Ortinski et al., 2010; Xie et al., 2010; Shigetomi et al., 2013a). Several AAV serotypes have been generated that show tropism preferentially for neurons or astrocytes or a combination of cells (Ortinski et al., 2010). The AAV 2/5 pseudotype shows the strongest tropism for astrocytes and therefore appears to have emerged as the AAV construct of choice for viral vector targeting of astrocytes. Substitution of the CMV minimal promoter with an astrocyte-specific promoter derived from GFAP results in astrocyte-specific expression of the transgene.

An example of an application of this technique in the study of astrocyte biochemistry and function is recording Ca^2+^ fluctuations in astrocytes. Monitoring astrocyte Ca^2+^ activity continues to be a primary technique in the study of astrocytes ever since it was discovered *in vitro* that astrocyte Ca^2+^ elevations modulate neuronal excitability (Parpura et al., 1994; Araque et al., 1998). Early approaches used commercially available membrane-permeable AM ester forms of organic Ca^2+^ indicator dyes to “bulk-load” and monitor changes in cytosolic Ca^2+^ concentration in astrocytes (Porter and McCarthy, 1996; Nett et al., 2002). This technique offered the advantage of monitoring Ca^2+^ activity in many astrocytes simultaneously, but at the cost of poor resolution of astrocyte processes that are the first responders to neuronal activity and which may operate autonomously from other astrocytic compartments. A derivative of this technique developed more recently is “bolus-loading” in which the AM Ca^2+^ indicator is pressure-ejected to load astrocytes with Ca^2+^ indicator deeper in the tissue where cells and their connections are more intact (Garaschuk et al., 2006). While this technique increases visibility of the larger astrocyte processes, it still suffers from low signal-to-noise and difficulty differentiating between domains of individual astrocytes due to high background labeling. Furthermore, a secondary label, such as sulforhodamine 101, is often required to confirm the loaded cells as astrocytes (Nimmerjahn et al., 2004), but this indicator has been shown to alter neuronal activity (Kang et al., 2010). Moreover, cell-impermeant Ca^2+^ indicators delivered via patch pipette offer excellent signal-to-noise and detection of Ca^2+^ events in small, bulk fine processes of astrocytes (Fiacco et al., 2007; Xie et al., 2012), but there is some concern about dialysis of the cellular contents by the patch pipette, especially in cases where the pipette is left in whole-cell patch clamp mode during measurements which can dampen Ca^2+^ signals. Last, while patch clamp delivery of Ca^2+^ indicator to astrocytes is relatively straightforward in brain slices, this approach is not as practical for recording astrocyte Ca^2+^ activity *in vivo*.

Viral-mediated delivery of GCaMP genetically encoded calcium indicators (GECIs) overcomes many of the limitations associated with the traditional approaches described above. It can be thought of as “high resolution bulk-loading” in that many astrocytes can be monitored at once and with the excellent signal-to-noise offered by the latest GCaMPs. Because the GECIs are delivered using an astrocyte-specific promoter, no secondary labeling is necessary to confirm the cells as astrocytes. Studies have indicated very specific transfection of astrocytes using this technique (Ortinski et al., 2010; Xie et al., 2010; Shigetomi et al., 2013a). Recently, Shigetomi et al. (2013a) used the AAV 2/5 vector to deliver a Lck membrane tethered form of GCaMP3 to astrocytes. Not only do the transfected astrocytes show significantly more frequent Ca^2+^ activity in fine processes compared to bulk-loading protocols (Shigetomi et al., 2013a), but because the indicator is membrane-tethered, it can monitor Ca^2+^ activity in the smallest “branchlets” and “leaflets” (Tong et al., 2013). Because Ca^2+^ activity monitored using the Lck-GCaMP3 indicator was diminished by an inhibitor of TRPA1 channels, the data provide evidence for astrocyte Ca^2+^ signals produced by a mechanism other than IP_3_R2-mediated internal stores. It will be important to follow-up this interesting finding in future studies by determining the relative ability of the Lck-GCaMP3 to detect TRPA1 vs*.* IP_3_R-mediated Ca^2+^ elevations or mitochondrial Ca^2+^ dynamics (Parnis et al., 2013) in astrocytes. For example, how much of the Ca^2+^ activity detected by Lck-GCaMP3 is diminished by IP_3_R inhibitors? This would help rule out possible non-specific effects of HC 030031, the drug used to block TRPA1 channels. Firmer evidence of the TRPA1-mediated Ca^2+^ elevations could be provided by transfecting IP_3_R2 KO mice with the Lck-GCaMP3 Ca^2+^ indicator and determining the extent to which any remaining Ca^2+^ elevations are blocked by the TRPA1 inhibitor. In summary, the membrane-tethered version of the new astrocytic GECIs may be specialized to distinguish among different sources of astrocyte Ca^2+^, due to a combination of its location, excellent signal-to-noise, and sensitivity to changes in Ca^2+^ concentration near the membrane.

Like any technique, viral delivery methods are not without limitations. GECIs have yet to be fully characterized. While they offer the advantage of increasing understanding of Ca^2+^ microdomains and conditions under which Ca^2+^ elevations occur, high levels of expression may result in significant calcium buffering with unintended consequences. Perhaps a more significant concern associated with the use of AAV vectors is the possibility of inducing reactive gliosis. Astrocytic Ca^2+^ signaling is disrupted in reactive astrocytes, with typically exaggerated and more widely propagating Ca^2+^ elevations and with increased propensity for gliotransmitter release (reviewed in Agulhon et al., 2012). Therefore, great care has been taken to determine the amount of reactive astrocytosis (if any) apparent after viral transfection by immunostaining for the astrocytic markers GFAP and vimentin to look for astrocytic hypertrophy (Xie et al., 2010; Shigetomi et al., 2013a). The AAV 2/5 vector was actually used purposefully to induce astrogliosis to determine the effect on astrocyte glutamine synthetase (GS) and neuronal excitability (Ortinski et al., 2010). Ortinski et al. (2010) found a significant increase in neuronal excitability caused by reduced GABA synthesis in neurons following loss of GS in reactive astrocytes. These data were confirmed by recovery of GABA transmission with exogenous application of glutamine. High titers of AAV 2/5 caused the astrogliosis and effects on neurons while low titers did not. Subsequent studies have actually used even higher titers than Ortinski et al. (2010), but reported normal astrocytic morphology suggesting that the astrocytes were not reactive (Xie et al., 2010; Shigetomi et al., 2013a). It is unclear what is behind these disparate findings but one possibility is that Ortinski et al. used the 638 bp GFAP Gfa104 promoter to target astrocytes, while Xie et al. (2010) and Shigetomi et al. (2013a,b) used the 681 bp GFAP gfaABC_1_D promoter. This is an important issue to continue to explore as there may be reactive changes induced by AAV in astrocytes that are underway prior to overt gliosis and hypertrophy. Because astrogliosis in itself significantly alters neuronal excitability, care needs to be taken interpreting the role of astrocyte Ca^2+^ on neuronal activity using virally transfected GECIs. Overall, the new astrocytic GECIs offer a tantalizing approach to record astrocyte Ca^2+^ activity *in vivo*. Future studies will help define limitations of viral-mediated GECI expression, leading to refinement in both methodology and interpretation of data.

### IN UTERO GENE ELECTROPORATION STRATEGIES

One emerging methodology to manipulate gene expression in astrocytes *in vivo* is by IUE. IUE is a method of gene delivery into mouse embryos (Fukuchi-Shimogori and Grove, 2001; Saito and Nakatsuji, 2001; Takahashi et al., 2002), which has become a method of choice for gain and loss of function studies in embryonic CNS cell progenitors. The IUE method involves injecting a plasmid DNA vector into the ventricles of the embryonic brain, and then using electrical pulses to facilitate the transfer of the DNA into the progenitor cells of the VZ/subventricular zone (SVZ). The optimal development stage for using this method in mice is between embryonic day (E) 10.5 and E16.5. In order to obtain stable expression of a transgene of interest in highly proliferative neural precursors and their progeny, a combination of transposon-mediated gene transfer into the host genome (Cary et al., 1989) with IUE has been used. This approach enables the expression of a transgene stably and efficiently in mitotic neural precursors (radial glia) during development, and in both cortical astrocytes and oligodendrocytes, after birth (Ding et al., 2005; Cadinanos and Bradley, 2007; Wilson et al., 2007; VandenDriessche et al., 2009; Woltjen et al., 2009; Yoshida et al., 2010; Chen and LoTurco, 2012). The transposon system involves a transgene of interest from a donor plasmid and a helper plasmid that expresses a transposase under the control of a cell-specific promoter (Cadinanos and Bradley, 2007; Chen and LoTurco, 2012). For instance, by combining a plasmid containing a transposase under the control of the GLAST promoter with a donor plasmid containing the green fluorescent marker eGFP, astrocytes originating from GLAST positive progenitors were labeled with eGFP in the cortex of P27 mice (Chen and LoTurco, 2012). Using an alternate transposon system and the GFAP and S100β promoters, stable postnatal expression of eGFP was obtained in astrocytes of juvenile mice (generated from GFAP- and S100β-expressing progenitors; Yoshida et al., 2010). The relatively low number of astrocytes expressing eGFP allowed the authors to trace the long-term lineage of glial progenitors *in vivo*.

Tracing the long-term lineage of glial progenitor is of great importance in the field because of the growing evidence for astrocytic morphological, molecular, and functional heterogeneity. Whether this heterogeneity is specified during brain development is not clear. A recent study has addressed this question and analyzed cell lineages thoroughly using IUE (Garcia-Marques and Lopez-Mascaraque, 2013). Twelve plasmids containing the sequences of six fluorescent proteins, whose expression was driven by the GFAP promoter in either the cytosol or the nucleus, were used. After co-electroporation of the plasmid mixture with a plasmid encoding a transposase under the control of the ubiquitous CMV promoter into ventricles of E14 embryos, the sequences coding the fluorescent markers were randomly inserted into the genome of progenitor cells to generate many colors by a combination of the fluorescent proteins. This stochastic and combinatorial expression of six fluorescent proteins produces inheritable markers for *in vivo* long-term tracing of glial progenitor lineages (Garcia-Marques and Lopez-Mascaraque, 2013). Unanticipated and highly specific clonal distribution in specific domains was revealed in the cortex of adult mice. Moreover, the authors found that different classes of astrocytes emerge from different clones, reinforcing the view that lineage origin defines astrocyte heterogeneity. The positional identity of these clones represents an additional level of astrocytic heterogeneity, which is likely associated with specific regional functions. Interestingly, astrocytes of the same clone often (but not always) responded equally to cortical injury, suggesting the dependence of their genetic information. While some clones exhibited strong morphological alteration following injury, other clones located similar distances from the lesion were unresponsive, suggesting that the developmentally determined features of different astrocytic clones should not be overlooked when developing brain therapies (Martin-Lopez et al., 2013).

In addition to the use of astrocyte-specific promoters to drive transgene expression in postnatal astrocytes, conditional CreER^T2^ systems have also been combined with IUE to induce cell- and time-specific expression of transgenes (Matsuda and Cepko, 2007; LoTurco et al., 2009), widening the set of possible genetic manipulations using IUE. Therefore, although the IUE technology has been used mainly for descriptive analysis so far, it is likely to become an important tool more commonly used in the field. The relative ease of implementation and inherent flexibility of a plasmid-based system should make this method valuable to many investigators interested in marking and manipulating glial progenitor lineages in the CNS of species for which Cre reporter lines are not available (e.g., rats). Additionally, one of the advantages of the IUE approach over the generation of transgenic mice is that it is both cheaper and less time consuming. Furthermore, compared to surgical viral transfer performed postnatally, it is less invasive. The introduction of transgenes in astrocytes through IUE, when the immune system is immature, does not produce overt reactive gliosis thereby enabling study of astrocytes in a more physiological context compared to viral-based approaches. However, limitations of IUE include variable electroporation efficiency, promoter leakiness, and difficulty in precisely controlling the number of labeled astrocytes.

## STUDYING ASTROCYTE FUNCTION THROUGH GENETIC MANIPULATION OF HUMAN GLIAL PROGENITORS

Although the molecular approaches described above have led to important steps forward in deciphering some of the functions of astrocytes in the pathophysiology of the mammalian brain, an important question remains: can the findings obtained from studies of the rodent brain be extrapolated to human astrocytes? There is mounting evidence that rodent astrocytes have multiple key functions in the developing and adult nervous system, including roles in synapse formation and function, working memory, autonomic and locomotor functions, and cognition (Barres, 2008; Halassa and Haydon, 2010; Lioy et al., 2011; Han et al., 2012; Molofsky et al., 2012; Wang et al., 2012a; Agulhon et al., 2013; Clarke and Barres, 2013). However, human astrocytes are both morphologically and functionally distinct from those of rodents (Colombo, 1996; Oberheim et al., 2009). Therefore, do human astrocytes have unique properties compared to their rodent counterparts, which could explain the higher cognitive abilities of humans? This fascinating question in the field has been recently explored by the groups of Maiken Nedergaard and Steven Goldman (Han et al., 2013).

In this study, the authors used human glial chimeric mouse brains to ask whether human astrocytes have unique properties that can influence activity-dependent synaptic plasticity *ex vivo* and learning and memory *in vivo*. To do so, they used human glial progenitor cells (GPCs) obtained by magnetic-assisted cell sorting using antibodies against PSA-NCAM and A5B5 cell surface antigens. The A2B5^+^/PSA-NCAM^-^ glial cells were expanded via a cell cultured protocol that promoted differentiation into astrocytes. Prior to transplantation into brains of neonatal immune-deficient mice, GPCs were transduced using a VSVg-pseudotyped lentiviral-CMC-EGFP in order to label them with a GFP. Once transplanted, mice matured to become adult chimeras for both mouse and human astrocytes (Windrem et al., 2004, 2008), and their brains were analyzed in adult mice. Human-derived cells survived in the rodent host brains and infiltrated the cortex and hippocampus to give rise to some EGFP^+^ astrocytes. Human astrocytes retained the large size and complex morphology that was previously reported in human brains (Oberheim et al., 2009), indicating that they matured in a cell-autonomous fashion. Additionally, human astrocytes extended processes contacting blood vessels and displayed long and tortuous processes, a phenotype typically observed in a specialized subpopulation of interlaminar astrocytes in the cortical white matter of adult human brains (Oberheim et al., 2009). Another type of engrafted human astroglial cell exhibited varicosity-studded processes (Kettenmann and Ransom, 2005; Oberheim et al., 2009). Finally, human astrocytes occupied distinct non-overlapping domains and formed gap junctions with rodent host astrocytes. At the physiological level, the input resistance of human astrocytes was twice as large as those of mouse astrocytes and Ca^2+^ signals propagated threefold faster than in mouse astrocytes. Notably, using acute hippocampal slices, Han et al. (2013) observed that the slope of fEPSPs was steeper and LTP was stronger and longer-lasting in mice grafted with human astrocytes compared to mice grafted with mouse astrocytes, indicating that excitatory synaptic transmission was enhanced in the presence of human astrocytes. Thus, enhancement of LTP was a specific characteristic of human glial cells. Interestingly, the potentiation of fEPSPs in human glial chimeric mice and the enhancement of LTP did not result from higher expression of NMDA receptors, increased synaptic release of glutamate, altered adenosine tone or increased glial release of D-serine. Rather it was found that human astrocytes facilitated synaptic insertion of the GluR1 subunit in host murine neurons through a TNFα-dependent, PKC/CaMKII-mediated pathway, consistent with the potentiation of AMPA receptor-mediated currents, thus lowering the threshold for induction of LTP in human glial chimeric mice. Strikingly, human glial chimeric mice performed better in hippocampus-mediated learning tasks compared to their untransplanted littermates. Together, these findings suggest that human astrocytes may have some unique properties to enhance cognition. This study represents an important scientific and technological step forward to study the function of human astrocytes in live adult brains, which has not been possible so far. It also illustrates the power of combining different technological advancements together in live animals. It was unclear in the study if varicose projection astrocytes were present only in cortical layers 5 and 6 as discovered previously in human temporal neocortex, or if these cells were present throughout the cortex and hippocampus in the chimeric mice. Abnormal expression of varicose projection astrocytes in the hippocampus may in part explain some of the unique cognitive abilities in these animals. In addition, further investigation will be needed to determine whether the enhanced cognitive performance of the humanized mice is specifically due to human astrocytes, or whether the progenitor cells that did not differentiate into mature astrocytes contributed to the phenotype.

## SUMMARY AND FUTURE DIRECTIONS

Important advances continue to be made in development of molecular tools to understand astrocyte function and the effect of manipulating individual astrocytic signaling molecules on neuronal activity and pathophysiology of the brain *in vivo*. Several examples have been provided in this review, including generation of conditional and inducible genetically engineered mouse lines for selective expression or removal of specific astrocyte proteins, as well as viral- or IUE-mediated gene delivery techniques for the study of astrocyte function. Study of astrocyte Ca^2+^ activity continues to be heavily emphasized, and while there is certainly more to discover in this area, there is an incredibly diverse array of other astrocytic GPCRs, ion channels, transporters, and signaling molecules to explore. Recent development of new lines using chemogenetics is one such example to further understand the importance of astrocytic signaling pathways in health and disease *in vivo*. The effect of selectively perturbing or stimulating other signaling molecules in astrocytes, such as G proteins, protein kinases, cAMP, diacylglycerol, phospholipase C, and phosphoinositol isoforms may open up new areas of research on astrocytes. Furthermore, a greater appreciation of astrocyte heterogeneity in different brain areas or even between adjacent synapses calls for the need to develop selective markers to identify astrocyte subtypes and molecular approaches to manipulate specific astrocyte subpopulations. Another recent molecular approach only just underway is understanding the effects of enhanced DNA methylation or de-methylation (epigenetic mechanisms) on astrocyte protein expression and function in development and disease. Finally, there has been a strong tendency to focus on the immediate, short-term consequences of manipulating astrocyte receptors and signaling molecules on neuronal activity and brain function. Based on the predominance of metabotropic signaling cascades in astrocytes and the role of astrocytes in homeostasis of the synaptic microenvironment, the role of astrocyte-to-neuron communication may be tuned toward long-term regulatory functions. Therefore, another area of future study is to examine the outcome of stimulating astrocytic signaling cascades on gene transcription, synthesis of new proteins, and long-term regulation of cellular processes in neurons, astrocytes, and other glial cell types.

## Conflict of Interest Statement

The authors declare that the research was conducted in the absence of any commercial or financial relationships that could be construed as a potential conflict of interest.
